# “Triangular ostectomy”: effective removal of bony interference during orthognathic surgery for better postoperative bone regeneration

**DOI:** 10.3389/froh.2026.1728503

**Published:** 2026-02-06

**Authors:** Hao Wu, Hongpu Wei, Qiao Long, Jian Cao, Hao Sun

**Affiliations:** 1 Department of Oral and Cranio-Maxillofacial Surgery, Shanghai Ninth People's Hospital, Shanghai Jiao Tong University School of Medicine, Shanghai, China; 2 College of Stomatology, Shanghai Jiao Tong University, Shanghai, China; 3 National Center for Stomatology, Shanghai, China; 4 National Clinical Research Center for Oral Diseases, Shanghai, China; 5 Shanghai Key Laboratory of Stomatology, Shanghai Research Institute of Stomatology, Shanghai, China; 6 Department of General Dentiatry, Dental and Ophthalmic Clinic of Putuo District, Shanghai, China

**Keywords:** bone regeneration, bony interference, orthognathic surgery, sagittal split ramus osteotomy (SSRO), virtual simulation

## Abstract

**Introduction:**

Sagittal split ramus osteotomy (SSRO) is commonly used to in orthognathic surgery especially in the treatment of mandible laterognathism. But frequently, distal and proximal mandible segments can't be aligned passively to another, which will impair bone regeneration after surgery. Herein, we developed a novel ostectomy method, named as “triangular ostectomy”, to remove bony interference fast and accurately, with the help of preoperative virtual planning and simulation.

**Methods:**

A total of 12 patients were involved. The progress of orthognathic was simulated before surgery. “Triangle ostectomy”, “posterior bending osteotomy” (PBO) technique and grinding method were applied. The patients' CT images were achieved 3 days and 4 months after surgery and the evaluation of alignment of proximal and distal segments was then carried out.

**Results and Discussion:**

After comparison, the “triangular ostectomy” was proved reliable to eliminate the bony interference quickly and accurately. The increased bone contact can accelerate postoperative bone regeneration. Furthermore, several tips during operation were also stated in this text to make this ostectomy easier and safer to operate.

**Conclusion:**

This “triangular ostectomy” technique to remove mandible bony interference should be taken into consideration to achieve better bone regeneration after SSRO, especially treating patient with severe facial asymmetry.

## 1 Introduction

One of the primary indications of orthognathic surgery is facial asymmetry, where mandible asymmetry is more obvious than maxilla. So far, the most widely applied surgical technique to treat mandible asymmetry is sagittal split ramus osteotomy (SSRO) (1, 15). This surgical technique allows a wide contact bony surface and an easy rigid fixation, so that patients can get a fast postoperative stabilization (3, 4).

Unfortunately, it is hard for distal and proximal segments to align themselves passively, especially in cases of asymmetry (5, 6). As for these patients, during the orthognathic surgery, it is always necessary to reposition the distal mandibular segment laterally and rotate the segment to the left or right side. This operation in turn can induce an anterior gap between segments and a posterior gap on the opposite side. It is usually inevitable that bony interference will appear between the distal and proximal segments. Sometimes, when the gap between the distal and proximal segments is large, a lateral bulge on patients' cheek can be clearly observed, even if a large piece of bone in proximal segment is cut off. Furthermore, the gap will decrease bone contact, which will in turn impair bone regeneration after surgery.

Due to these bony interferences, the proximal mandibular segment is going be displaced during internal fixation, leading to the displacement of condyle, which will increase the risk of early relapse after orthognathic surgery (7, 8). Furthermore, in the long term, the condylar torque caused by displacement of proximal segment can consequently increase the incidence of temporomandibular disorders. Afterwards, doctors have developed modified techniques based on conventional SSRO to overcome these shortcomings, such as the short lingual osteotomy (SLO) technique and the distal cutting technique (9, 10).

In 2007, in order to remove bony interference more efficiently, Ellis reported a novel osteotomy technique to treat the distal segment, where an additional vertical osteotomy was performed behind the terminal molar (11). Using this technique, almost all bony interferences could be effectively eliminated, even though the patients exhibited severe asymmetry. Just after the lateralization of the inferior alveolar nerve (IAN), this vertical osteotomy could be performed fully or partially with a fissure bur or saw. Later, the ultrasonic and piezoelectric bone cutting device was used to avoid the injury of IAN and soft tissue (2, 12). With a piezoelectric device, almost all the cortical bone could be cut thoroughly and thus the posterior segment could be easily fractured. Afterwards, position screws were applied to fixing the posterior segment to the proximal mandibular segment. This widely-used osteotomy technique for removal of bony interferences was called as “posterior bending osteotomy” (PBO).

However, in clinical practice, PBO techniques are time-consuming and will prolong surgical time. And during vertical osteotomy, the risk of IAN damage still exists and should be carefully avoided, which requires more surgical experience. Herein, for a safer and faster technique to remove bony interference, we developed a novel ostectomy method, named as “triangular ostectomy”. In this study, we compared this new technology with traditional grinding method and PBO technique to reveal the feasibility and efficiency of “triangular ostectomy”.

## 2 Materials and methods

### 2.1 Involved patients

A total of 12 patients from July 2022 to March 2023 with skeletal mandibular laterognathism were involved, who accepted orthognathic surgery at the Department of Oral and Maxillofacial Surgery, Ninth People's Hospital. This study has been approved by the ethics committee of Shanghai Ninth People's Hospital (SH9H-2021-T30-2). The studies were conducted in accordance with the local legislation and institutional requirements. Since it is retrospective study, written informed consent for participation was not necessarily required from the participants or the participants' legal guardians/next of kin in accordance with the national legislation and institutional requirements. However, as a routine practice in the department, the patients and legal guardians/next of kin were still required to provide written informed consent before surgery.

### 2.2 Preoperative simulation

**Figure 1:**
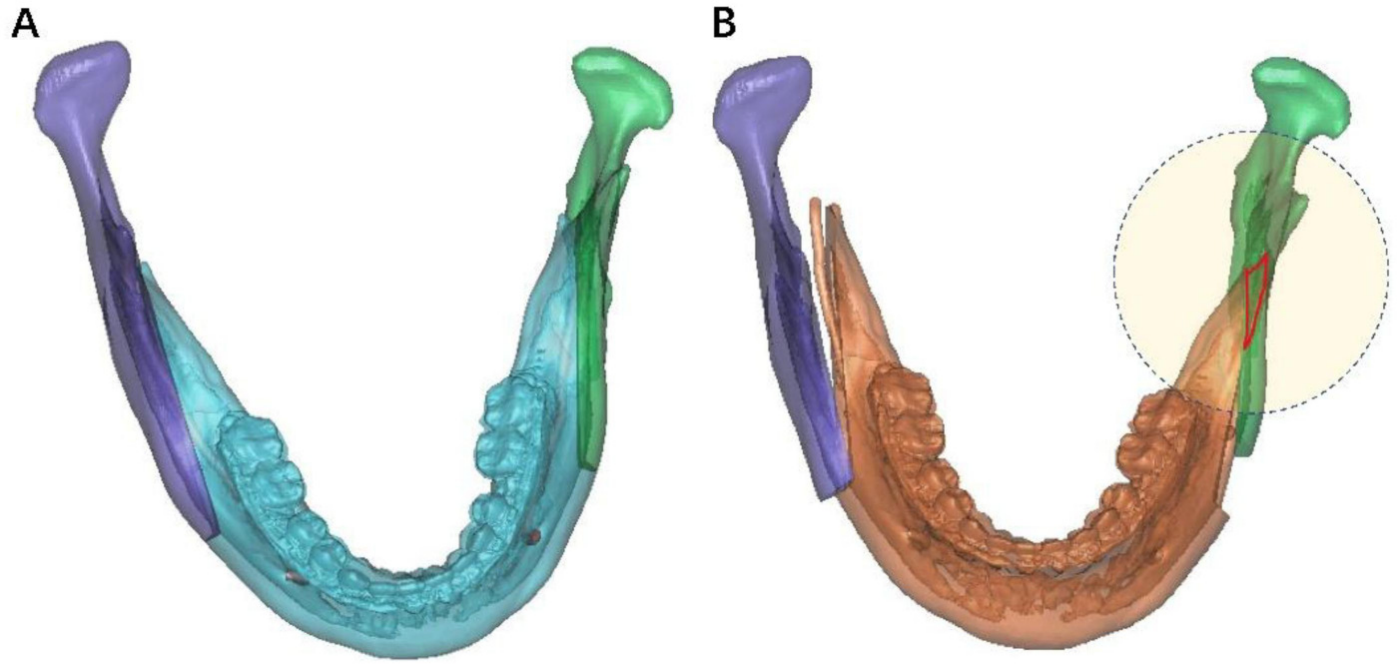
3D virtual simulation of SSRO to treat mandible retrognathism. **(A)** Proximal and distal segments before surgery. **(B)** Proximal and distal segments after surgery. The red region in circle represents the bony interference.

The CT images were reconstructed as three-dimensional (3D) models using Proplan CMF 3.0. The progress of orthognathic was simulated before surgery and the shape of the bony interference was determined which usually exhibited triangular shape in the top view (Figure 1).

### 2.3 Surgical procedures

All SSRO surgeries were performed using the modified Hunsuck technique. Early contact between the inner and outer bone plates occurred in all cases after sagittal osteotomy. Among them, eight cases used the “triangle ostectomy” technique to remove bony interference. The steps are listed as follows: (1) The shape of the bony interference was confirmed by preoperative 3D simulation (2) The distal mandibular segment was pulled outward and rotated clockwise to fully expose the position of the osteotomy line on the inner side of the distal segment; (3) A tunnel retractor was placed deep behind the inner side of the distal segment to fully expose the lingula and protect the inferior alveolar nerve (IAN); (4) Below the horizontal osteotomy line, a triangular bony interference on the lingual side was removed by saw; (5) The final occlusal splint was placed, and after maxillomandibular fixation with stainless wires, the distal and proximal segments were aligned passively in close contact and fixed with a titanium plate. One case used the modified Ellis technique to eliminate the early contact. A vertical osteotomy was made in the distal part of the proximal segment, just behind second molar, and the inner side was “greenstick” fractured. During the operation, the IAN needed to be dissected for adequate protection. Three cases used the traditional grinding method with bur, where the inner part of the proximal segment was grinded to remove the bony interference for passive alignment. The operating time was recorded.

### 2.4 Postoperative evaluation

The patients' CT images were achieved 3 days and 4 months after surgery. The evaluation of alignment of proximal and distal segments was then carried out based on the 3D reconstruction of CT images.

## 3 Results and discussion

**Figure 2:**
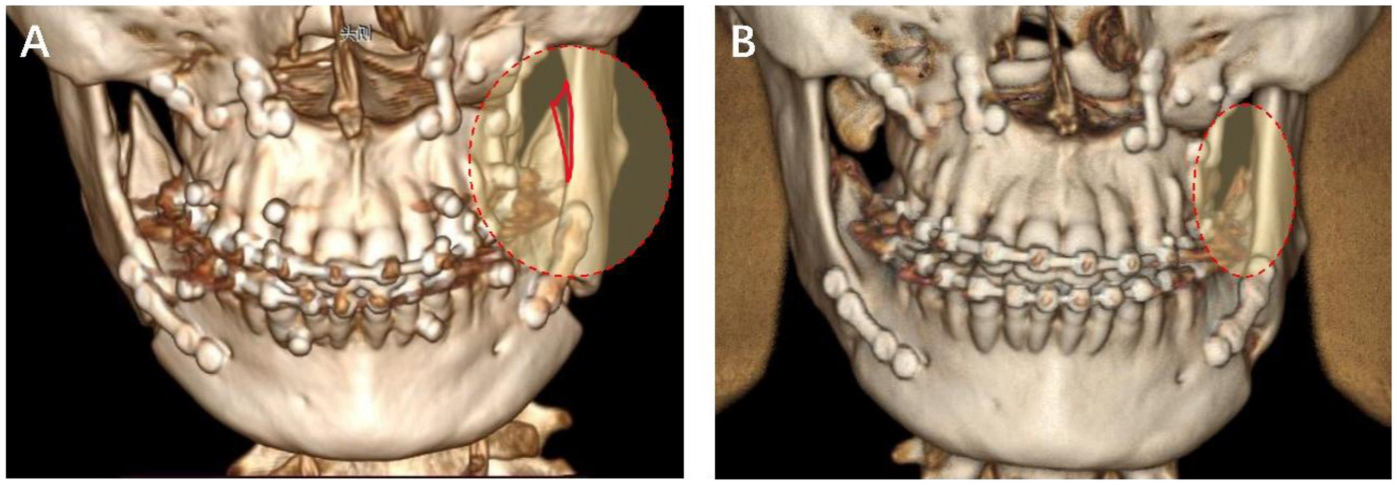
3D reconstruction of CT images post orthognathic surgery. **(A)** 3 days after surgery. The bony interference in red region was completely removed and the proximal and distal segments were aligned passively. **(B)** 4 months after surgery. Bone regeneration could be obviously observed in the red region.

After treated by “triangle ostectomy” technique, grinding method and PBO technique, the bony interference between distal and proximal segments was completely removed, which aligned themselves passively to one another, allowing more bone contact for better bone regeneration postoperatively (Figure 2A). And after 4 months, obvious new bone regeneration could be observed, where the gap between distal and proximal segments disappeared (Figure 2B). The results indicated the promoted bone regeneration after “triangular ostectomy”, since the contact area of proximal and distal segments was significantly increased after passive alignment. The passive alignment of proximal and distal segments could do great favor to post operative bone regeneration, which could in turn accelerate patients' recovery and postoperative functionalization of mandible. The increased contact area of proximal and distal segments played an important role in this progress. Furthermore, the average operation time for “triangle ostectomy” technique in 8 cases was 4.2 min; where PBO technique cost about 15 min and traditional grinding method cost about 8 min. The “triangle ostectomy” technique significantly reduced surgical times and got an accurate removal of bony interference successfully.

**Figure 3:**
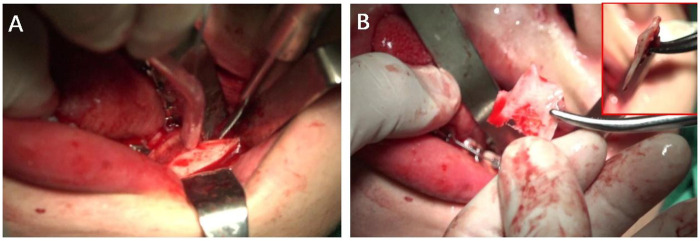
Photos during operation of “Triangular ostectomy”. **(A)** The assistant pull the distal segment outwardly to the biggest extent and tunnel retractor was placed deep behind the inner side of the distal segment to fully expose the lingula and protect the IAN. **(B)** The removed bony interference.

Compared with Ellis' PBO technology, the biggest difference of “Triangular ostectomy” was the vertical osteotomy near the mandibular lingula to remove interference directly, while PBO technology actually created “greenstick” fracture after osteotomy. The efficiency of “triangle ostectomy” technique could be much better and the risk of unfavorable fracture could be decreased. Notably, the “triangle ostectomy” technique exhibited the advantage of better protection of IAN. During ostectomy, the assistant was required to pull the distal segment outwardly to the biggest extent after bilateral sagittal split, so that IAN could be fully exposed. And under the protection of tunnel retractor, the cutting of the interfered part by reciprocating saw could be much safer (Figure 3).

3-dimentional (3D) simulation has been applied in the prediction of bony interference during SSRO for several years, which can give guidance of bone removal (13, 14). In our clinical practice, 3D simulation was also of great importance. The removed part of proximal segment could be accurately presented via virtual simulation. Notably, when move proximal segment, the condyle should be seated in the fossa. Otherwise, the mispositioning of condyle may cause TMJ complications and increase the risk of relapse.

During operation, there were several tips for faster and safer removal of bony interference. (1) If the posterior part of maxilla needs a large descending, the ramus will probably be occluded, which will affect the visual field, and hinder the flap procedure. To avoid this situation, before operating on maxilla, the mandibular flap can be degloved previously. And the mandible osteotomy can be completed in advance without splitting. (2) The best chance for “triangle ostectomy” technique is the time just after bilateral sagittal split was completed, when the distal segment can be moved to the biggest extent. At this point, the bony interference can be easily exposed, and the ostectomy can be much safer. (3) During ostectomy, the tunnel retractor should be placed deeply behind the distal segment to fully expose the lingula and protect the IAN. (4) When cutting off the interfered part by reciprocating saw, the assistant should pull the distal segment outwardly and rotate it clockwise for better exposure.

However, there still exist some problems of this technique. If not estimated accurately, the excess or insufficient removal of bone may happen, which will prolong operation time. And after excess bone was removed, a gap will appear between proximal and distal segments, where the effusion can be accumulated in this space, and thus cause infection. Therefore, after excessive ostectomy, post operative pressing is recommended. Meanwhile, despite the protection of assistant, the IAN still face the risk of injury, especially the volume of bony interference is oversized. Under this situation, the grinding method should be applied combined with “triangular ostectomy” to improve the efficiency and safety of bony interference removal.

## 4 Conclusion

The surgical modification proposed in this study, which involves performing a “triangular ostectomy” below the lingual, can quickly and accurately eliminate the bony interference of proximal and distal segments, which can allow more bone contact and promote bone regeneration postoperatively. Especially treated patient with severe facial asymmetry, this technique to remove bony interference should be taken into consideration.

## Data Availability

The original contributions presented in the study are included in the article/Supplementary Material, further inquiries can be directed to the corresponding author.
